# To break the cycle of COVID-19 lockdowns and safely open up economies, we must ensure equitable access to diagnosis and treatment

**DOI:** 10.4102/ajlm.v11i1.1650

**Published:** 2022-05-27

**Authors:** Nqobile Ndlovu, Yenew K. Tebeje, Renuka Gadde, Trevor Peter

**Affiliations:** 1 African Society for Laboratory Medicine, Addis Ababa, Ethiopia; 2 Africa Centres for Disease Control and Prevention, Addis Ababa, Ethiopia; 3 Clinton Health Access Initiative, Boston, Massachusetts, United States

In June 2021, Sub-Saharan Africa was facing the worst crisis from coronavirus disease 2019 (COVID-19) since the pandemic began, with cases and deaths rising amidst the emergence of new virulent strains of severe acute respiratory coronavirus 2.^1^ In the midst of this, countries need to ramp up effective prevention and treatment programmes in a way that permits economies to function. These measures include vaccination, physical distancing and wearing masks to protect others, testing and tracing to identify and isolate infected individuals and those who have been in contact with them, and treatment with emerging drug therapies in order to reduce transmission and save lives.^2^

There is optimism and hope in the global discussion on vaccination and novel therapies. However, effective COVID-19 disease control is likely to require that countries jointly balance their investments in vaccines, personal protection, testing and treatment.^3,4^ Testing programmes which have been a backbone of control efforts since the onset of the pandemic, now need to be integrated effectively with other interventions as well as continually improve their efficacy. For example, vaccine rollout must be supported by testing and surveillance data to improve outcomes. The use of routine testing data can help to identify transmission hot spots and to prioritize locations and populations within vaccination campaigns.^5^ Testing data will be important for monitoring the occurrence of outbreaks within populations post-vaccination and to inform ongoing efforts to close residual gaps in coverage, including informing governments on how to develop targeteted approaches for emerging viral variants.^5^ Testing is also needed for the effective use of emerging drug therapies and medical oxygen. These rely on diagnosis and treatment early in the course of disease to achieve the highest benefits in morbidity and mortality.^4^ In the near future, the combination of rapid tests and new drug regimens will allow the expansion of test and treat programmes that will make COVID-19 a manageable infection.

In the meantime, to operate schools and workplaces safely and sustainably, it is important to ensure that rapid testing is available where and when it is needed and that we have the tools in place to comprehensively monitor and track the disease.^6^ Current testing efforts in many sub-Saharan countries are well below the threshold of one test per 1000 population per week set by the World Health Organization.^7^ This means that not only is it not clear how many infections have occurred, but there is limited insight into the emergence and spread of new outbreaks. There are ongoing efforts to better understand vaccine variants through genomic sequencing but there are limitations. For example, sequencing technologies are still not widely available and they often do not provide real-time information on variant outbreaks to help limit spread.^8^

Without the ability of individuals and public health programmes to understand their infection status and take actions limit disease spread, the current cycle of COVID-19-related restrictions on work, school and travel will continue to threaten Africa’s economies, increasing poverty and hunger, and reversing gains made in recent decades. That is why it is critical for the global health community, governments, and the private sector to come together to ensure that accurate, rapid antigen diagnostic tests (RDTs) are accessible at community level, including self-tests. Rapid antigen diagnostic tests must be available in all primary health care facilities and made available through public and private channels for self testing, and must be appropriately used to help reopen borders, schools, and places of work and worship.^9,10^

Examples of this work were presented in a recent series of webinars organised by the African Society for Laboratory Medicine and the Africa Centers for Disease Control and focused on expanding access to antigen RDTs for COVID-19.^9^ Leading scientists and public health experts from Africa, Europe, the United States and Asia presented the latest updates on testing and COVID-19 control. In Rwanda, COVID-19 RDTs are being used in public and private health facilities, at weddings, schools, prisons, churches, market places, as well as for refugees and others recommended by Rwanda’s Ministry of Health. This has helped treble the number of testing locations and reduce testing costs. In South Africa, RDTs have been widely used in open border posts (land, sea, and air) and are being used within the provinces of the country. In Nigeria, scaled-up use of RDTs within National Youth Camps has made it possible to quickly detect cases and isolate youth as they entered the camps, thus preventing potential outbreaks.^9^

Schools provide one of the best opportunities for testing and other strategies to prevent transmission. The most important lines of defence in preventing transmission of severe acute respiratory coronavirus 2 include making sure students do not come to school with symptoms, enforcing universal mask adherence and physical distancing, improving ventilation, and swiftly investigating any cases associated with schools, including contact tracing and quarantine and isolation.^6^ Testing is another tool in the prevention arsenal for schools and can provide guidance on how to plan better prevention and increase layers of defense. Testing also ensures early identification of cases among students and staff and helps with contact tracing efforts, and can also identify infection among students and staff at high-risk of developing severe disease due to underlying conditions and support investigations and studies in understanding the role children play in disease transmission.

Testing must reflect the purposes and resources of a community. Sweeping guidance that schools should be closed if the positivity rate exceeds a certain threshold needs to be reviewed in the context of a country. For example, if a school is conducting all in-person classes and the prevalence in community increases, it may consider reducing class sizes and having students attend on alternating schedules. In other words, a deeper analysis should guide decision making. There is mounting evidence of the detrimental effects of school closures and the impact it is having on children from learning loss to emotional wellness.^10^ One can argue that schools should be in the same category as essential services, similar to healthcare facilities, in that they should only close when there is no other option.

In September 2020, several organisations, including the World Health Organization, The Global Fund, the Africa Centres for Disease Control and Prevention, FIND and others, announced an agreement to make available 120 million low-cost, high-quality antigen RDTs for COVID-19 for low- and middle-income countries.^11^ While this was necessary to ensure that tests were available, adoption and use of antigen testing was slow, and many countries implemented these professional-use tests widely within health facilities and at the community level (i.e., for symptomatic and high-risk individuals). High-performing self-tests are now more widely available and provide opportunities to expand access to testing, decongest health facilities, and limit the risk of transmission.^12^ It is important that countries identify the most effective use for both professional use rapid tests and self tests to be deployed within public testing programmes and for private use.^13^

Now is not the time to debate whether we should be spending on vaccines, oxygen, personal protective equipment, drug treatment, or testing. Effective programmes need to strategically deploy each of these interventions in an integrated way, just as integrated prevention, testing and treatment control programmes have been developed for other diseases. Fortunately, antigen RDTs with excellent performance are available and can be used at the point of care or in laboratory settings, and are increasingly available for self testing.^12,14^ Even though much progress has been made in recent years, gaps in access to essential tests remain an issue throughout Africa, including COVID-19. While robust systems to deliver testing and surveillance for COVID-19 are developed, it is important to ensure that they are sustainable into the future and also address other major health issues, including malaria, HIV, tuberculosis, cervical cancer and other diseases. Maintaining a joint focus on prevention, testing and treatment will be necessary, building on partnerships between governments, donors, policymakers and industry to use the available tools to solve this global crisis.
